# Acute Pharmacological Effects and Oral Fluid Biomarkers of the Synthetic Cannabinoid UR-144 and THC in Recreational Users

**DOI:** 10.3390/biology10040257

**Published:** 2021-03-24

**Authors:** Nunzia La Maida, Esther Papaseit, Lucia Martínez, Clara Pérez-Mañá, Lourdes Poyatos, Manuela Pellegrini, Simona Pichini, Roberta Pacifici, Mireia Ventura, Liliana Galindo, Francesco Paolo Busardò, Magí Farré

**Affiliations:** 1 Department of Excellence of Biomedical Science and Public Health, University “Politecnica delle Marche” of Ancona, Via Tronto 71, 60124 Ancona, Italy; n.lamaida@pm.univpm.it (N.L.M.); f.p.busardo@staff.univpm.it (F.P.B.); 2 Clinical Pharmacology Unit, Hospital Universitari Germans Trias i Pujol and Institut de Recerca Germans Trias i Pujol (HUGTiP-IGTP), 08916 Badalona, Spain; epapaseit.germanstrias@gencat.cat (E.P.); lucia.mdsoto@gmail.com (L.M.); cperezm.mn.ics@gencat.cat (C.P.-M.); lpoyatos@igtp.cat (L.P.); mfarre.germanstrias@gencat.cat (M.F.); 3 Department of Pharmacology, Therapeutics and Toxicology, Universitat Autònoma de Barcelona, 08193 Cerdanyola del Vallés, Spain; 4 Clinical Phamacology Department, Hospital Universitario La Paz, 28046 Madrid, Spain; 5 National Centre on Addiction and Doping, Istituto Superiore di Sanità, V.Le Regina Elena 299, 00161 Rome, Italy; simona.pichini@iss.it (S.P.); roberta.pacifici@iss.it (R.P.); 6 Energy Control, Associació Benestar i Desenvolupament, 08041 Barcelona, Spain; mireia@energycontrol.org (M.V.); lg532@cam.ac.uk (L.G.); 7 Department of Psychiatry, University of Cambridge/Cambridgeshire and Peterborough NHS Foundation Trust, Cambridge CB20QQ, UK

**Keywords:** UR-144, synthetic cannabinoids (SCs), cannabis, THC, physiological effects, subjective effects, oral fluid concentration

## Abstract

**Simple Summary:**

UR-144 is a synthetic cannabinoid found in herbal incenses for recreational use as a substitute of cannabis. It is a cannabinoid receptor agonist with effects on the central nervous system similar to those of THC. Several cases of intoxication involving UR-144 consumption have been reported. An observational study was carried out to assess UR-144 acute pharmacological effects in comparison with cannabis measuring biomarkers of disposition in oral fluid. Both UR-144 and THC increased blood pressure and heart rate. THC induced stimulant-like and high effects significantly more than those of UR-144 and the two parent drugs could be measured in oral fluid as biomarkers of consumption within 3 h following smoking of the substance.

**Abstract:**

Synthetic cannabinoids (SCs) are one of the most frequent classes of new psychoactive substances monitored by the EU Early Warning System and World Health Organization. UR-144 is a SC with a relative low affinity for the CB1 receptor with respect to that for the CB2 receptor. As with other cannabinoid receptor agonists, it has been monitored by the EU Early Warning System since 2012 for severe adverse effects on consumers. Since data for UR-144 human pharmacology are very limited, an observational study was carried out to evaluate its acute pharmacological effects following its administration using a cannabis joint as term of comparison. Disposition of UR-144 and delta-9-tetrahydrocannibinol (THC) was investigated in oral fluid. Sixteen volunteers smoked a joint prepared with tobacco and 1 or 1.5 mg dose of UR-144 (*n* = 8) or cannabis flowering tops containing 10 or 20 mg THC (*n* = 8). Physiological variables including systolic and diastolic blood pressure, heart rate and cutaneous temperature were measured. A set of Visual Analog Scales (VAS), the Addiction Research Centre Inventory (ARCI)-49-item short form version and the Evaluation of the Subjective Effects of Substances with Abuse Potential (VESSPA-SSE) were administered to evaluate subjective effects. Oral fluid was collected at baseline, 10, 20, 40 min and 1, 2, 3 and 4 h after smoking, for UR-144 or THC concentration monitoring. Results showed significant statistical increases in both systolic and diastolic blood pressure and heart rate after both UR-144 and cannabis smoking. Both substances produced an increase in VAS related to stimulant-like and high effects, but scores were significantly higher after cannabis administration. No hallucinogenic effects were observed. Maximal oral fluid UR-144 and THC concentrations appeared at 20 and 10 min after smoking, respectively. The presence of UR-144 in oral fluid constitutes a non-invasive biomarker of SC consumption. The results of this observational study provide valuable preliminary data of the pharmacological effects of UR-144, showing a similar profile of cardiovascular effects in comparison with THC but lower intensity of subjective effects. Our results have to be confirmed by research in a larger sample to extensively clarify pharmacological effects and the health risk profile of UR-144.

## 1. Introduction

Cannabis is the most commonly used illegal psychotropic drug, primarily consumed for recreational purposes [1,2]. There are more than 480 identifiable chemical constituents known in the cannabis plant and about 85 different cannabinoids have been isolated, Δ^9^-tetrahidrocannabinol (THC) being the psychoactive one [3]. It is known that THC primarily acts as a partial agonist on two cannabinoid receptors CB1 and CB2, mediated by the G-protein-coupling. CB1 receptors are located mainly in neurons of the central and peripheral nervous system [4]. The primary effect of cannabinoids in these receptors is the inhibition of synaptic transmission, which causes changes of mood and perception, such as pain sensation, sleep, body temperature or food intake [5]. Cannabinoids also target CB2 receptors, located in tissues of the immune system. The CB1 and CB2 receptors can also be activated by substances secreted by our bodies, the endogenous cannabinoids or endocannabinoids, anandamide and 2-arachidonoylglycerol [6].

In recent decades, scientists have synthesized and tested in vitro different compounds to study CB1/CB2 binding activity [7]. From 2006 these substances, defined as synthetic cannabinoids (SCs), or “synthetic cannabinoid receptor agonists” (SCRAs) and commonly called “Spice”, have been misused as a replacement for cannabis-like effects in European and other countries, becoming popular from around 2008 [8], so that the “Spice phenomenon” appeared as a legal alternative for cannabis [9]. Soon, some European authorities banned these compounds, but, as a result, modified chemicals came into this new market, with many similar “Spice-like” products. The number of different identified SCs was more than 300 from 2008 to 2020 [10]. They are characterized by their high affinity to CB1 receptors and their interaction with other non-cannabinoid receptors, presenting different effects and risk profiles [2,11]. 

One of the most popular SCs is UR-144 ([(1-pentyl-1H-indol-3-yl)(2,2,3,3-tetramethylcyclopropyl)-methanone]. It was synthetized in 2006 by Abbott Laboratories, first reported in herbal incenses seized in June 2012 in Korea and has spread very quickly all over the world as a substitute for cannabis, similarly to other SCRAs [12]. After its detection in numerous herbal products marketed under a variety of names, UR-144 has been banned in many countries [13]. Usually UR-144 is smoked in “joints”, but it can also be taken orally, vaporized or inhaled [14]. The metabolism of UR-144 has not been systematically studied, but available data show that is extensively metabolized by CYP3A4 at the tetra-methyl-cyclopropyl moiety with minor contributions of CYP1A2. Consequently, concomitant use of CYP3A4 inducer or inhibitor drugs could influence the kinetics and effects and could produce potential drug–drug interactions [15]. 

The most characteristic symptoms described in cases of UR-144 consumption, analytically confirmed, included slurred speech and dilated pupils, poor coordination, unsteady gait and difficulty standing, abnormal pupillary reaction, cheerful behavior, poor coordination and staggering, less frequent verbosity, narrow pupils, loss of consciousness, pale or reddened facial skin, blackout, euphoria, agitation, hallucinations, hindered communication, shaking hands, seizures, convulsions, somnolence, delayed movements, redness of the conjunctiva, and tachycardia [16]. Additionally, UR-144 and metabolites have been widely detected in multiple polydrug intoxication driving under the influence cases [17,18,19,20,21,22]. 

As usual and for several different SCs, despite its high prevalence on the drug market and its implication in severe intoxications, there is a of lack human studies on UR-144 pharmacodynamics and pharmacokinetics under controlled administration.

We set up and carried out an observational study to evaluate the acute pharmacological effects, as well as biomarkers of time course kinetics, of smoked UR-144 in consumers, in comparison with smoked cannabis as a reference. Our null hypothesis was that both cannabinoids would produce similar pharmacological effects but those following smoked cannabis would be more intense. Due to the nature of observational study in a naturalistic non-clinical setting, oral fluid (OF) was collected as non-invasive biological matrix to assess disposition of UR-144 and THC in relation to pharmacological effects.

## 2. Materials and Methods

### 2.1. Participants

A total of sixteen healthy polydrug recreational users, who had reported previous multiple experience with cannabis and having used SCs at least once in their lives, were enrolled for the study (13 males and 3 females). Exclusion criteria were a history of any serious medical or psychopathological disorder including substance use disorder (except nicotine), a previous serious adverse reaction with cannabis or SCs, and chronic medicine use. Participants were recruited by word-of-mouth and snowball sampling through the harm reduction non-governmental organization, Energy Control. The study protocol was submitted and approved by the Clinical Research Ethics Committee of our center, the Hospital Universitari Germans Trias i Pujol (CEI HUGTiP, Badalona, Spain; ref. PI-18-267). The investigation was conducted according to the Declaration of Helsinki recommendations and Spanish laws concerning clinical research. All the participants were correctly and fully informed of the purpose, methods and means of the study. All indicated their agreement to participate and signed an informed consent prior to inclusion. Participants received a financial compensation for their participation.

### 2.2. Study Design and Treatments

The design of this study was a naturalistic, prospective, observational study, with minimal intervention in recreation drug users who self-administered one dose of UR-144 or cannabis (with 15%THC and less than 0.1% cannabidiol) by smoking a joint in which the substances were mixed with tobacco. The joint was inhaled for 5 min following the subject’s usual form (5–12 inhalations), and the mouth was washed-out with plain water to reduce contamination that could interfere with OF sampling.

All the doses that were self-administered were also self-selected by each participant, based presumably on their previous experience. Subjects brought the substances to the testing site themselves, which they had obtained from an unknown source. Although no information was available about the synthesis of the drug, similar products tested by Energy Control, a harm reduction organization that provides a Drug Checking Service for users, showed that the substance contained SCs at 95% purity with no toxic adulterants. The UR-144 and THC contents had been previously analyzed by means of gas chromatography associated with mass spectrometry (GC/MS). The method used permits checking for most common drugs of abuse including cocaine, MDMA, LSD, amphetamine and methamphetamine, heroin, 2C-B and other phenethylamines, DMT and other tryptamines, ketamine, psilocybin, salvinorin A, natural and synthetic cannabinoids, and most of the new psychoactive substances [23].

The dose of UR-144 was selected after reviewing the literature [14,15,16,17,18]. The WHO Expert Committee on Drug Dependence (Thirty-ninth Meeting, Geneva, 6–10 November 2017) [14], reported that the starting dose range is reported by users as 0.5–2 mg. Similar doses were reported in users of the substance by Energy Control in its harm reduction activities and they recommended subjects to take 1–1.5 mg to avoid possible health risks. The mean dose of UR-144 was 1.25 mg (four males self-administered a dose of 1.5 mg, three males and one female self-administered 1 mg) and the mean dose of THC in the self-administered cannabis was 18.75 mg (six males and one female self-administered a dose of 20 mg and one male self-administered 10 mg, calculated as amount of cannabis containing 15%THC).

### 2.3. Procedures

Before the study sessions all participants underwent a general medical examination and a psychiatric evaluation.

They received training with respect to the questionnaires and procedures employed in the study. Sessions took place at a private club with ambient music and participants could talk, read, or play table games during the session and interact, with the exception of the evaluation time. Each study session was done on a different day. On the day of the session, subjects were admitted to the selected recreational venue and they were asked about any event that could affect their participation. They were asked to abstain from any drug use for two days prior the session and alcohol concentrations in expired air were measured before the beginning of the sessions.

Urine spot samples were collected prior to the administration, to exclude drug use (benzodiazepines, barbiturates, morphine, cocaine, amphetamines, methamphetamines, MDMA, marijuana, phencyclidine) with One Step Rapid Test 10 Test Drug Screen (Gima, Gessate, Milan, Italy).

Assessments were performed at baseline (pre-dose), at 10, 20 and 40 min, and at 1, 2, 3 and 4 h after self-administration of UR-144 and cannabis.

### 2.4. Physiological Effects

Non-invasive systolic blood pressure (SBP) and diastolic blood pressure (DBP) and heart rate (HR) were determined with an Omron^®^ monitor (Barcelona, Spain) at baseline, 10, 20 and 40 min, and 1, 2, 3 and 4 h after administration. Oral temperature was measured at the same time.

### 2.5. Subjective Effects

Subjective effects were measured with visual analogue scales (VAS), the 49-item Addiction Research Center Inventory form (ARCI) and the Evaluation of Subjective Effects of Substances with Abuse Potential questionnaire (VESSPA). A set of different VAS [100 mm (mm)] labeled with different adjectives marked at opposite ends with “not at all” and “extremely” were employed [24,25]. Subjects were asked to rate effects such as “intensity”, “high”, “good effects”, “bad effects”, “hunger”, “drowsiness”, “dizziness”, “confusion”, “nausea”, “vomits”, “anxiety”, “aggressiveness”, “hallucinations-seeing of lights or spots”, “hallucinations-hearing sounds or voices” and “hallucinations-seeing animals, things, insects or people”.

The Spanish validated version of the short-form ARCI is a true/false 49-item questionnaire, an instrument for the determination of subjective drug effects. It includes five subscales related to: drug sedation (pentobarbital-chlorpromazine-alcohol group, PCAG), euphoria (morphine-benzedrine group, MBG), dysphoria and somatic symptoms (lysergic acid diethylamide group, LSD), intellectual efficiency and energy (benzedrine group, BG) and d-amphetamine-like effects (amphetamine, A) [26].

The VESSPA is a questionnaire that enables the measurement of changes in subjective effects caused by different drugs, including stimulants and psychedelics. It includes six subscales: sedation (S), psychosomatic anxiety (ANX), changes in perception (CP), pleasure and sociability (SOC), activity and energy (ACT), and psychotic symptoms (PS) [27].

The visual analogue scales were administered at baseline, 10, 20 and 40 min, and at 1, 2, 3 and 4 h after drug administration. ARCI and VESSPA forms were completed at baseline, and at 1, 2, 3 and 4 h after drug administration.

Adverse events were assessed throughout the study session and were reported within 24-h after the self-administration session (by a phone call). 

### 2.6. Drugs Disposition in Oral Fluid

OF was collected with Salivette^®^ tubes (Barcelona, Spain) to determine the concentrations of UR-144 and THC at baseline, 10, 20 and 40 min, and 1, 2, 3 and 4 h after self-administration. Samples were centrifuged and frozen at −20 °C until posterior analysis by a modified and validated liquid chromatography-mass spectrometry method to quantify THC and UR-144, but permitting detection of metabolites (LC-MS/MS) [28,29].

### 2.7. Statistical Analysis

Sample size was determined based on the methodology of bioequivalence studies (seven-eight subjects would be needed considering an alpha risk of 0.05, a power of 80%, 20% variability and an increase in subjective effect intensity of at least 30% from cannabis to UR-144).

For physiological and subjective variables, differences with respect to baseline were calculated. Peak effects (E_max_) were determined and the area under the curve of the effects (AUC_0–4h_) was calculated using the trapezoidal rule by the Pharmacokinetic Functions for Microsoft Excel (Usansky, Desai and Tang-Liu, Department of Pharmacokinetics and Drug Metabolism, Allergan, Irvine, CA, USA).

E_max_ and AUC_0–4h_ between UR-144 and THC were compared using a Student’s *t*-test for unpaired sample. Differences in time to reach peak effects (T_max_) values were assessed using a Non-Parametric Test (Wilcoxon test).

To compare the time course (T-C) of effects between UR-144 and THC, a one-factor repeated measures ANOVA (baseline, 10, 20, 40 min and 1, 2, 3 and 4 h) was performed. Furthermore, a Dunnett multiple comparison post hoc test was conducted to evaluate the effects along time for UR-144 and THC comparing the different time points with baseline (times 0–10 min, 0–20 min, 0–40 min, 0–1 h, 0–2 h, 0–3 h and 0–4 h). Statistically analyses were performed using PAWS Statistics version 18 (SPSS Inc., Chicago, IL, USA). Statistically significance was defined as *p* < 0.05.

For UR-144 and THC oral fluid concentrations, C_max_, T_max_ and the AUC_0–4h_ were calculated using the Pharmacokinetic Functions for Microsoft Excel.

## 3. Results

### 3.1. Participants

The sixteen healthy subjects recruited for the study were polydrug recreational users who reported previous multiple experience with cannabis and had used SCs at least once in their lives. Most of the subjects have experience with psychostimulants and hallucinogens.

Eight subjects consumed UR-144 by smoking a self-made joint (seven males and one female). Four males self-administered a dose of 1.5 mg while three males and one female self-administered 1 mg substance mixed with tobacco. They had a mean age of 28 ± 7 years (range: 23–41 years), weighed 70.4 ± 6.80 kg (range: 62–83 kg), and their mean body mass index (BMI) was 21.96 ± 1.67 kg/m^2^ (range: 18.12 ± 23.15 kg/m^2^). All reported past month marijuana use (17.80 ± 10.01 days, range 1–28) and six were current tobacco smokers. 

Another eight subjects, five males and two females, self-administered a dose of 20 mg, and one male self-administered 10 mg THC smoking a cannabis joint. They had a mean age of 31 ± 8 years (range: 23–42 years), weighed 64.8 ± 8.88 kg (range: 48–75 kg), and their mean body mass index (BMI) was 21.06 ± 2.63 kg/m^2^ (range: 17.88 ± 25.31 kg/m^2^). All of them reported past month marijuana use (17.00 ± 11.22 days, range 4–30) and current tobacco use.

### 3.2. Physiological Effects

**Figure 1:**
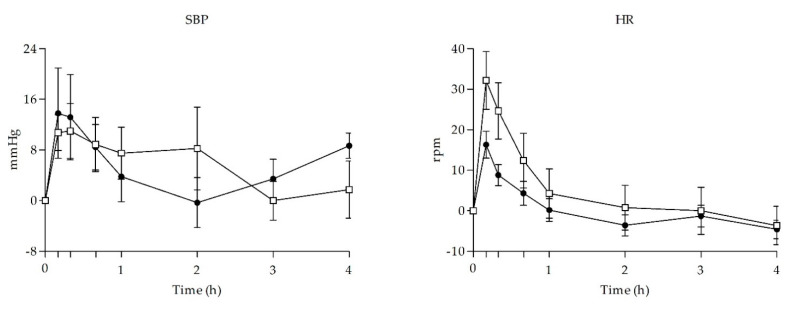
Time course of main physiological signs observed after self-administration of UR-144 (*n* = 8) and THC (*n* = 8). Values are differences from baseline. Symbols: ● UR-144, □ THC; values are mean and standard error. SBP = systolic blood pressure; HR = heart rate.

Acute effects of UR-144 and THC smoking on physiological variables are presented in Table 1 and time course (TC) effects of the main variables are shown in Figure 1.

In comparison to baseline levels, UR-144 and THC smoking produced statistically significant increase in diastolic blood pressure (DBP) and heart rate (HR) and UR-144 also in systolic blood pressure (SBP). When comparing UR-144 and THC, no statistically significant differences were found in terms of peak effects (E_max_), area under the curve from 0 to 4 h (AUC_0–4h_) and/or TC points.

### 3.3. Subjective Effects

**Figure 2:**
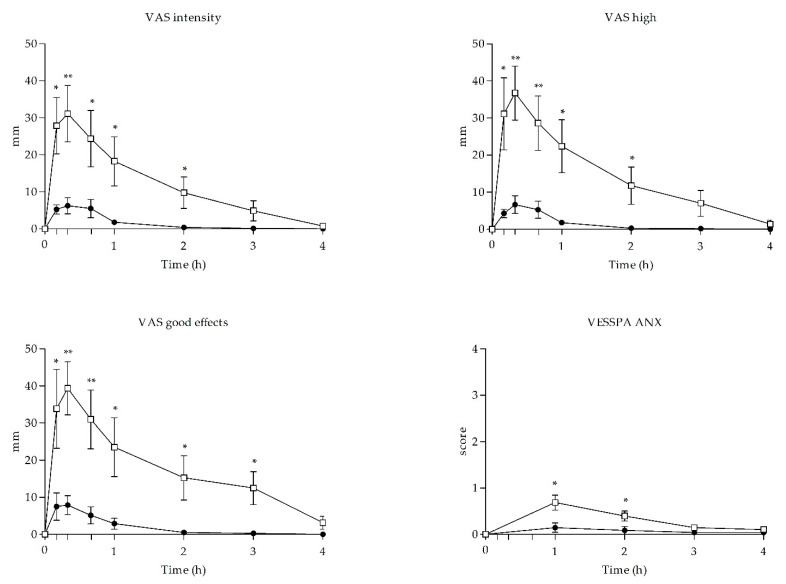
Time course of subjective effects observed after self-administration of UR-144 (*n* = 8) and THC (*n* = 8). Values are differences from baseline. Symbols: ● UR-144, □ THC; values are mean and standard error. SBP = systolic blood pressure; HR = heart rate. * statistically significant a *p* < 0.05; ** statistically significant a *p* < 0.01.

Acute effects of UR-144 and cannabis smoking on subjective variables are presented in Table 1 and time course effects of the main significant variables shown in Figure 2.

Self-administration of a single dose of UR-144 and THC increased the scores of stimulant-like effects and euphoria measured by visual analog scales (VAS). Compared to baseline levels, UR-144 and cannabis smoking produced statistically significant increases for VAS ‘stimulated’, ‘high’, and ‘good effects’ and VAS ‘hunger’. Significant difference between UR-144 and cannabis smoking were detected in E_max_, AUC_0–4h_ and TC points for VAS ‘stimulated’, ‘high’, and ‘good effects’. For the rest of the VAS, no statistically significant changes were detected after self-administration of UR-144 or THC.

In relation to the Addiction Research Center Inventory (ARCI) questionnaire, UR-144 and cannabis did not induce significant increases of scores compared to baseline with the exception to UR-144 for euphoria subscale (MBG: morphine-benzedrine group).

Regarding the effects measured with the Evaluation of Subjective Effects of Substances with Abuse Potential (VESSPA-SA) questionnaire, significant changes with respect to baseline were detected for psychosomatic anxiety (ANX), changes in perception (CP) and psychotic symptoms (PS) subscales after UR-144 self-administration and for activity and energy (AE) and PS subscales after cannabis self-administration. Differences between UR-144 and THC in E_max_, AUC_0–4h_ and TC points were detected for ANX. 

In reference to adverse events, all the selected doses were well tolerated with no relevant adverse events during the study session. Within 24 h after the start of the smoking session, none of the subjects reported adverse effects.

### 3.4. UR-144 and THC of Concentrations

After UR-144 smoking, OF concentrations increased quickly, reaching a peak concentration at 20 min (min) after self-administration (time to reach peak effects (T_max_)). Concentrations decreased rapidly from 20 min to 1 h after administration and could be detected in oral fluid for up to 2 h. At 3 h, subjects presented undetectable concentrations or concentration <0.4 ng/mL. Maximal concentration (C_max_) was 14.85 ± 4.77 ng/mL and AUC_0–4h_ was 12.62 ± 2.78 ng·h/mL. 

After cannabis smoking, OF THC concentrations reached its peak concentration at 10 min (T_max_) with a mean value of 2.09 ± 2.42 mg/dL (C_max_) and concentrations decreased slowly until 1 h after administration. AUC from 0 to 4 h was 1.29 ± 0.73 ng·h/mL. No eventual UR-144 or THC metabolites were detected in OF.

**Figure 3:**
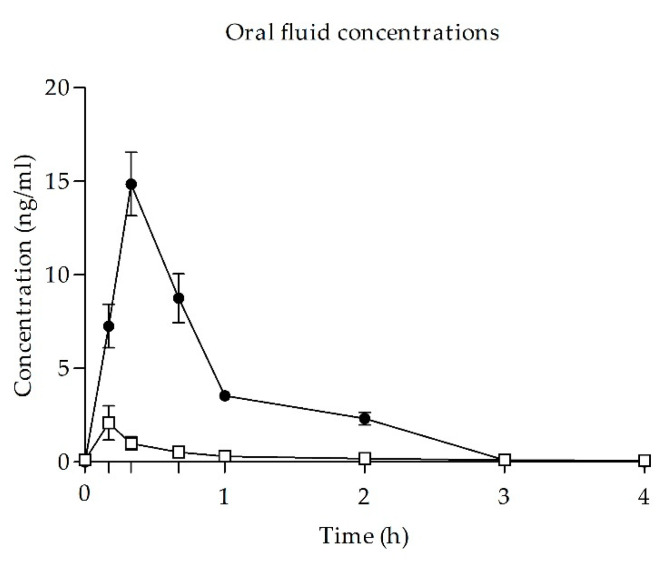
Time course of UR-144 (*n* = 8) and cannabis (*n* = 8) concentrations in oral fluid. Symbols: ● UR-144; □ THC; values are mean and standard error.

The OF concentration-time curves for UR-144 and THC are shown in Figure 3.

## 4. Discussion 

Cannabis is the most commonly used illegal drug in the world and its use is increasingly present in our society both for its recreational (natural cannabis and synthetic cannabinoids) and medical applications. In recent times medical cannabis shows an increasing use, with preparations with higher concentrations of cannabidiol, a natural cannabinoid that does not show reinforcing and abuse properties [7,30,31]. Cannabis use can produce short and long-term health consequences [7,32]. Reports suggest that severe adverse reactions and fatal intoxications are much more common with SCs than with cannabis.

To the best of our knowledge, the current study is the first attempt to assess in a non-controlled setting the acute pharmacology and biomarkers of disposition of UR-144 in recreational users’ OF.

The preliminary results obtained point out that self-administration by inhalation route of a single recreative dose of UR-144 induces prototypical cannabimimetic effects. Thus, in a non-controlled setting the profile of physiological effects produced by UR-144 is characterized by moderate increases of blood pressure (SBP and DBP) and HR, similarly to those produced by inhaled cannabis. The onset of cardiovascular effects occurred at 10 min after smoking and was maintained over a short-lasting period of 1 h. The acute subjective effects of UR-144 consist of euphoria and well-being, but significantly lower in magnitude and duration than those observed for cannabis. This finding is in accordance with the literature that describes approximately a two-fold lower affinity of UR-144 to the CB1 receptor as compared to THC [33]. In fact, the low psychotropic influence induces the use of high doses that can lead to unexpected and potential toxic effects. Additionally, subjects who smoked UR-144 experienced an increase in appetite and somnolence similarly to those who smoked cannabis. In relation to bad effects, dizziness or confusion, minimal scores were reported after UR-144 consumption.

In reference to cannabis effects, those observed in our study were those prototypically described after the inhaled administration of doses in the administered range, with clear increases in cardiovascular parameters, and feelings of well-being and high [34]. Cannabis produced higher scores than UR-144 in most of the evaluated effects.

Our results are the first data on UR-144’s pharmacokinetic profile reported in OF. Maximal OF concentrations of UR-144 were reached at 10 min following substance smoking, similarly to OF peak concentrations reported following JWH-122 and JWH-210 smoking [35] in analogous naturalistic conditions. In addition, measured OF concentrations matched with single measurements of OF UR-144 in forensic and intoxication cases, ranging from <5–30 ng/mL [36]. It is notable to highlight that in urine and blood samples of UR-144 consumers, the detection of the parent drug is less frequent than that of its metabolites since the parent compound is present only in the first hours after consumption. In this respect, the results presented provide valuable analytical data to be considered in toxicological cases. The concentrations of THC in OF were also in the range of those described in controlled studies conducted in our clinical trials unit (1.29 ng/mL) [30].

The present study has several intrinsic limitations due to its naturalistic-observational design (non-placebo-controlled design), the small sample size, the unknown origin of the substance, the limited number of time-point measures and the lack of blood and/or other biological matrices collection. However, there are a number of strengths to remark: the participation of males and females previously experienced with inhaled cannabis and SCs, self-selection of real-life recreational dosages by the subjects according to their preferences, cannabis as a well-known active comparator, the real recreational setting and the use of validated methodology and analytic techniques.

In summary our results showed that UR-144 exhibits the prototypical effects of THC on heart rate and blood pressure but less subjective effects such as intensity or high. THC showed a relatively good tolerability as described previously [34]. We did not find relevant toxic effects of UR-144, probably due to the relative low dose administered, but in other studies this substance has been associated with severe side effects (16).

## 5. Conclusions

We have here presented preliminary data on the acute pharmacological and health risk profile of UR-144 in comparison to cannabis and, consequently, THC use. The administration of UR-144 in naturalistic conditions resulted in detectable concentrations of the substance in OF as a biomarker of consumption in this non-invasive biological matrix.

More research in controlled studies with a wider range of doses and including larger samples is needed to better define the human pharmacology of UR-144 and to facilitate its comparison with THC and other SCs.

## Figures and Tables

**Table 1:** Summary of significant statistical result on acute physiological and subjective effects observed after administration of UR-144 (*n* = 8) and cannabis (*n* = 8).

	Parameter	Mean ± SD		T StudentComparison Effects UR-114 vs. THC		ANOVAComparison Time-Course Effects UR-144 vs. THC		
		UR-144	THC	t	*p*-Value	F	*p*-Value	T-C points
SBP (mmHg)	E_max_	14.94 ± 22.92	17.63 ± 17.74	−0.262	0.797			
	AUC_0–4h_	18.36 ± 34.25	21.59 ± 39.64	−0.175	0.864			
	TC					1.100	0.370	--
DBP (mmHg)	E_max_	11.56 ± 10.15	15.31 ± 5.82	−0.906	0.380			
	AUC_0–4h_	17.02 ± 22.53	28.74 ± 25.77	−0.969	0.380			
	TC					1.867	0.083	--
HR (beats/min)	E_max_	10.81 ± 17.37	24.13 ± 31.59	−1.044	0.314			
	AUC_0–4h_	−0.74 ± 26.86	17.27 ± 62.54	−0.748	0.467			
	TC					3.902	**0.001**	NS
T (°C)	E_max_	−0.38 ± 0.44	0.10 ± 0.46	−0.611	0.551			
	AUC_0–4h_	0.26 ± 0.98	0.33 ± 1.18	−0.117	0.908			
	TC					1.747	0.207	--
Intensity	E_max_	8.13 ± 6.22	39.88 ± 16.76	−5.023	**<0.001**			
	AUC_0–4h_	5.92 ± 5.73	47.62 ± 42.33	−2.761	**0.015**			
	TC					4.945	**<0.001**	10, **20**, 40, 1, 2
High	E_max_	8.75 ± 5.78	43.75 ± 20.99	−4.548	**<0.001**			
	AUC_0–4h_	5.63 ± 5.16	58.16 ± 48.61	−3.039	**0.009**			
	TC					6.520	**<0.001**	10, **20**, **40**, 1, 2
Good effects	E_max_	11.38 ± 10.23	46.62 ± 21.71	−4.154	**0.001**			
	AUC_0–4h_	7.56 ± 7.53	70.68 ± 56.91	−3.110	**0.008**			
	TC					5.180	**<0.001**	10, **20**, **40**, 1, 2, 3
Bad effects	AUC_0–4h_	1.25 ± 2.37	5.00 ± 6.39	−1.555	0.142			
	TC	0.84 ± 1.98	3.25 ± 5.26	−1.215	0.244			
	T-C					0.841	0.556	--
Hunger	E_max_	35.37 ± 26.92	30.37 ± 29.87	0.352	0.730			
	AUC_0–4h_	68.46 ± 70.43	55.89 ± 66.79	0.366	0.722			
	TC					0.470	0.854	--
Somnolence	E_max_	20.37 ± 23.42	11.50 ± 13.20	0.934	0.366			
	AUC_0−4_	48.81 ± 78.76	22.52 ± 32.00	0.875	0.397			
	T-C					0.618	0.740	--
Dizziness	E_max_	3.50 ± 6.09	4.00 ± 3.46	−0.202	0.843			
	AUC_0–4h_	1.15 ± 1.89	3.11 ± 4.67	−1.099	0.290			
	TC					1.469	0.187	--
Confusion	E_max_	2.00 ± 4.11	3.26 ± 5.01	−0.546	0.594			
	AUC_0–4h_	0.58 ± 0.90	1.05 ± 1.69	−0.687	0.503			
	TC					1.054	0.399	--
Nausea	E_max_	1.37 ± 2.72	0.12 ± 0.35	1.288	0.219			
	AUC_0–4h_	0.28 ± 0.45	0.21 ± 0.06	1.606	0.131			
	TC					0.517	0.993	--
Vomit	E_max_	0.13 ± 0.35	0.00 ± 0.00	1.000	0.334			
	AUC_0–4h_	0.03 ± 0.09	0.00 ± 0.00	1.000	0.334			
	TC					1.000	0.436	--
Anxiety	E_max_	4.63 ± 12.68	3.50 ± 8.40	0.209	0.837			
	AUC_0–4h_	1.99 ± 5.53	1.84 ± 4.46	0.060	0.953			
	TC					0.149	0.994	--
Aggressiveness	E_max_	1.25 ± 3.15	0.00 ± 0.00	1.122	0.281			
	AUC_0–4h_	0.00 ± 0.00	0.00 ± 0.00	1.178	0.259			
	TC					1.299	0.259	--
Hallucinations-seeing of lights or spots	E_max_	0.36 ± 0.86	0.00 ± 0.00	0.858	0.405			
	AUC_0–4h_	0.50 ± 1.41	0.66 ± 1.22	0.243	0.812			
	TC					0.758	0.623	--
Hallucinations-hearings of sounds or voices	E_max_	0.00 ± 0.00	0.00 ± 0.00	--	--			
	AUC_0–4h_	0.00 ± 0.00	0.00 ± 0.00	--	--			
	TC					--	--	--
Hallucinations-seeing animals, things, insects, or people	E_max_	0.13 ± 0.35	0.00 ± 0.00	1.000	0.334			
	AUC_0–4h_	0.08 ± 2.37	0.00 ± 0.00	1.000	0.334			
	TC					1.000	0.436	--
PCAG	E_max_	2.63 ± 1.60	4.63 ± 2.27	−2.041	0.061			
	AUC_0–4h_	5.50 ± 4.82	8.50 ± 6.88	−1.011	0.329			
	TC					1.342	0.266	--
MBG	E_max_	1.00 ± 1.93	2.63 ± 2.39	−1.498	0.156			
	AUC_0–4h_	2.69 ± 5.99	5.94 ± 6.04	−1.080	0.298			
	TC					2.331	0.067	--
LSD	E_max_	−0.88 ± 1.13	0.63 ± 2.97	−1.335	0.203			
	v	−1.31 ± 1.28	1.06 ± 4.81	−1.351	0.198			
	T-C					3.973	**0.007**	10
BG	E_max_	0.88 ± 1.55	0.13 ± 2.42	0.739	0.472			
	AUC_0–4h_	0.44 ± 0.98	1.38 ± 4.49	−0.577	0.573			
	TC					2.507	0.052	--
A	E_max_	1.63 ± 2.39	2.25 ± 1.98	−0.570	0.578			
	AUC_0–4h_	3.38 ± 5.26	5.56 ± 5.29	−0.829	0.421			
	TC					0.874	0.485	--
S	E_max_	0.63 ± 0.52	0.75 ± 0.49	−0.487	0.633			
	AUC_0–4h_	1.43 ± 1.41	1.44 ± 0.97	−0.019	0.985			
	TC					2.709	**0.039**	NS
ANX	E_max_	0.15 ± 0.29	0.69 ± 0.46	−2.837	0.013			
	AUC_0–4h_	0.29 ± 0.69	1.28 ± 0.82	−2.596	**0.021**			
	TC					6.154	**<0.001**	1, 2
CP	E_max_	0.02 ± 0.06	0.08 ± 0.13	−1.269	0.225			
	AUC_0–4h_	0.02 ± 0.60	0.08 ± 0.13	−1.269	0.225			
	TC					3.444	**0.014**	NS
SOC	E_max_	0.77 ± 0.97	0.96 ± 1.13	−0.361	0.724			
	AUC_0–4h_	1.49 ± 2.61	2.63 ± 3.61	−0.721	0.483			
ACT	E_max_	0.31 ± 0.56	0.25 ± 0.25	0.288	0.777			
	AUC_0–4h_	0.53 ± 0.89	0.54 ± 0.58	−0.030	0.977			
	TC					1.066	0.382	NS
PS	E_max_	0.11 ± 0.24	0.06 ± 0.89	0.463	0.650			
	AUC_0–4h_	0.18 ± 0.44	0.64 ± 0.09	0.721	0.483			
	TC					0.409	0.801	NS

E_max_ = peak effects 0–4 h (differences from baseline); AUC_0–4h_ = Area under the curve from 0 to 4 h; T-C = temporal course from 0 to 4 h. For E_max_ and AUC_0–4h_, a Student’s *t*-Test for independent sample was used (see Statistical Analysis). A *p*-value < 0.05 (bold) was considered statistically significant. For T-C, a one-factor repeated measures ANOVA was used to measured differences between UR*−*144 and THC. Statistical differences are and presented as “time point” *p* < 0.05, in bold when “time point” *p* < 0.01.

## Data Availability

The data presented in this study are available on request from the corresponding author.
